# Non-neural Muscle Weakness Has Limited Influence on Complexity of Motor Control during Gait

**DOI:** 10.3389/fnhum.2018.00005

**Published:** 2018-01-31

**Authors:** Marije Goudriaan, Benjamin R. Shuman, Katherine M. Steele, Marleen Van den Hauwe, Nathalie Goemans, Guy Molenaers, Kaat Desloovere

**Affiliations:** ^1^Department of Rehabilitation Sciences, University of Leuven, Leuven, Belgium; ^2^Clinical Motion Analysis Laboratory, University Hospitals Leuven, Pellenberg, Belgium; ^3^Mechanical Engineering, University of Washington, Seattle, WA, United States; ^4^WRF Institute for Neuroengineering, University of Washington, Seattle, WA, United States; ^5^Department of Child Neurology, University Hospitals Leuven, Leuven, Belgium; ^6^Department of Development and Regeneration, University of Leuven, Leuven, Belgium; ^7^Department of Orthopedics, University Hospitals Leuven, Pellenberg, Belgium

**Keywords:** cerebral palsy, Duchenne muscular dystrophy, motor control, muscle synergies, gait analysis, muscle weakness

## Abstract

Cerebral palsy (CP) and Duchenne muscular dystrophy (DMD) are neuromuscular disorders characterized by muscle weakness. Weakness in CP has neural and non-neural components, whereas in DMD, weakness can be considered as a predominantly non-neural problem. Despite the different underlying causes, weakness is a constraint for the central nervous system when controlling gait. CP demonstrates decreased complexity of motor control during gait from muscle synergy analysis, which is reflected by a higher total variance accounted for by one synergy (tVAF_1_). However, it remains unclear if weakness directly contributes to higher tVAF_1_ in CP, or whether altered tVAF_1_ reflects mainly neural impairments. If muscle weakness directly contributes to higher tVAF_1_, then tVAF_1_ should also be increased in DMD. To examine the etiology of increased tVAF_1_, muscle activity data of gluteus medius, rectus femoris, medial hamstrings, medial gastrocnemius, and tibialis anterior were measured at self-selected walking speed, and strength data from knee extensors, knee flexors, dorsiflexors and plantar flexors, were analyzed in 15 children with CP [median (IQR) age: 8.9 (2.2)], 15 boys with DMD [8.7 (3.1)], and 15 typical developing (TD) children [8.6 (2.7)]. We computed tVAF_1_ from 10 concatenated steps with non-negative matrix factorization, and compared tVAF_1_ between the three groups with a Mann-Whiney *U*-test. Spearman's rank correlation coefficients were used to determine if weakness in specific muscle groups contributed to altered tVAF_1_. No significant differences in tVAF_1_ were found between DMD [tVAF_1_: 0.60 (0.07)] and TD children [0.65 (0.07)], while tVAF_1_ was significantly higher in CP [(0.74 (0.09)] than in the other groups (both *p* < 0.005). In CP, weakness in the plantar flexors was related to higher tVAF_1_ (*r* = −0.72). In DMD, knee extensor weakness related to increased tVAF_1_ (*r* = −0.50). These results suggest that the non-neural weakness in DMD had limited influence on complexity of motor control during gait and that the higher tVAF_1_ in children with CP is mainly related to neural impairments caused by the brain lesion.

## Introduction

Two of the most common neurological and neuromuscular diseases in childhood are cerebral palsy (CP) and Duchenne muscular dystrophy (DMD) (Sussman, 2002; Rosenbaum et al., 2007; Graham et al., 2016). CP is defined as “a group of permanent disorders of the development of movement and posture, causing activity limitations attributed to non-progressive disturbances that occurred in the developing fetal or infant brain” (Rosenbaum et al., 2007). DMD is characterized by an altered gene on the X-chromosome, which codes for the protein dystrophin. Lack of dystrophin in muscles leads to a disbalance between damage and repair of the muscle fibers (Kobayashi and Campbell, 2012). This damage results in muscles that predominantly consist of fat and fibrous tissue (Sussman, 2002; Jones et al., 2010). Although CP and DMD have different origins and expressions, they have at least one symptom in common: muscle weakness. In both groups, muscle weakness is considered an important contributor to their pathological gait patterns (Sutherland et al., 1981; D'Angelo et al., 2009; Gage et al., 2009; Gaudreault et al., 2010; Doglio et al., 2011; Ganea et al., 2012; Steele et al., 2012).

There are only a small number of studies describing DMD gait (Sutherland et al., 1981; D'Angelo et al., 2009; Gaudreault et al., 2010; Doglio et al., 2011; Ganea et al., 2012; Ropars et al., 2016). Furthermore, none of these studies have verified to what extent these gait deviations were associated with muscle weakness (Sutherland et al., 1981; D'Angelo et al., 2009; Gaudreault et al., 2010; Doglio et al., 2011; Ganea et al., 2012; Ropars et al., 2016). In CP, several researchers have analyzed the relationship between muscle weakness and gait impairments, but their results are inconsistent (Damiano et al., 1995, 2010; Wiley and Damiano, 1998; Desloovere et al., 2006; Lee et al., 2008; Dallmeijer et al., 2011; Eek et al., 2011; Sagawa et al., 2013; Meyns et al., 2016; Shin et al., 2016). In DMD, muscle weakness is caused by non-neural changes in muscle morphology, whereas in CP, muscle weakness has neural as well as non-neural components. Neural components are considered the primary cause of weakness and are the result of the original brain injury (Gage et al., 2009). Examples include altered motor unit recruitment patterns and decreased selective motor control (Gage et al., 2009; Mockford and Caulton, 2010). Non-neural components are considered secondary causes of muscle weakness in CP (Gage et al., 2009), including changes in muscle morphology or lever-arm dysfunction due to bony deformities (Gage et al., 2009; Barrett and Lichtwark, 2010). The effect of these neural and non-neural changes on gait can be very different than their effect on strength assessments, such as maximal voluntary isometric contractions (MVICs). This could be an important reason for the discrepancies in previous studies analyzing the relationship between muscle weakness and altered gait in CP.

Despite the lack of consensus on how muscle weakness contributes to impaired gait, there is no doubt that it is a constraint the central nervous system (CNS) needs to deal with when initiating and controlling gait. Since children with CP and boys with DMD have different etiologies of weakness, the evaluation of how the CNS copes with muscle weakness in both populations has the potential to increase our understanding of the relationship between muscle weakness and gait deviations. In particular, it will help to differentiate between the relative effects of both neural and non-neural components of weakness on gait.

The regulation of human gait is not entirely understood, largely due to the abundant degrees of freedom (DOFs) and complexity of the human body (Latash, 2012). One of the theories for how humans control this abundance, is the use of muscle synergies instead of individual control of each muscle. Muscle synergies have been defined as the “consistent patterns of multi-muscle coordination that generate specific action” (Ting et al., 2015). Synergistic patterns of muscle recruitment have been well documented during various rhythmic tasks, including walking (d'Avella et al., 2003; Dietz, 2003; Nielsen, 2003; Barroso et al., 2013). Central pattern generators in the spinal cord and supra-spinal structures are thought to contribute to the regulation of these synergistic muscle activations (Lacquaniti et al., 1999; Dietz, 2002, 2003; Nielsen, 2003; Petersen et al., 2012). Synergies are flexible, thereby allowing to compensate for internal and external disturbances without affecting the outcome of the intended movement (Latash et al., 2002; Ting et al., 2015). This suggests that neural as well as non-neural components can affect synergies (Kutch and Valero-Cuevas, 2012; Bizzi and Cheung, 2013; Clark, 2015). In synergy analysis, evaluating the “total variance accounted for” (tVAF) by a given number of synergies can quantify the complexity of an individual's muscle activation patterns during dynamic tasks. The tVAF by one synergy (tVAF_1_) can provide a summary measure of synergy complexity. When tVAF_1_ is high, one synergy can explain a large part of the variance in muscle activity, representing a decrease in complexity of motor control during the analyzed task (Steele et al., 2015; Ting et al., 2015). Individuals with a CNS motor lesion, such as in CP or stroke, have higher tVAF_1_ during gait than age-related healthy controls (Clark et al., 2010; Clark, 2015; Steele et al., 2015; Tang et al., 2015; Ting et al., 2015). Further, this decrease in complexity of motor control in children with CP was found to be related to muscle weakness (Steele et al., 2015). However, in these prior studies, muscle weakness was measured via a global summary score from manual muscle testing (MMT) (Steele et al., 2015). Not only does MMT have low reliability in young children with developmental disabilities (Mahony et al., 2009), but these analyses also limit our understanding of whether weakness of specific muscles affects control of gait.

If muscle weakness is a constraint for the CNS, it could limit the available degrees of freedom (DOFs) and negatively influence complexity of motor control, not only in CP, but also in boys with DMD. In children with CP, there is one confounding factor: the influence of the brain lesion on synergies and muscle weakness. Alterations in the CNS, such as the brain lesion in CP, affect a substantial part of synergy regulation (Lacquaniti et al., 1999; Dietz, 2002, 2003; Nielsen, 2003; Petersen et al., 2012). This brain lesion also underlies muscle weakness (its neural component) (Gage et al., 2009; Mockford and Caulton, 2010). The relationship between muscle weakness and tVAF_1_ found in the previous study (Steele et al., 2015) could be caused by their mutual underlying source: the alterations in the CNS. This poses the research question: is muscle weakness contributing to higher tVAF_1_ during gait in children with CP or is the higher tVAF_1_ a quantification of the underlying brain lesion?

The primary goal of this research was to compare and contrast the impact of muscle weakness on tVAF_1_ extracted from synergy analysis during gait for children with CP and DMD. We evaluated tVAF_1_ during gait at self-selected walking speed for three groups of children: children with CP, children with DMD, and TD children. We expected decreases in complexity of motor control (increase in tVAF_1_) in both CP and DMD children when compared to a control group of TD children. However, in children with CP, due to the addition of a neural component of muscle weakness, a higher tVAF_1_ was expected than in DMD. As a secondary goal, we also sought to analyze the effect of muscle weakness of four muscle groups (knee extensors, knee flexors, plantar flexors, and dorsiflexors) on complexity of motor control. Muscle weakness was assessed via MVICs with a standardized protocol, using a hand-held dynamometer (HHD) in a fixed position (Goudriaan et al., 2018). We hypothesized that of the four measured muscle groups, the plantar flexors would be largely responsible for higher tVAF_1_ in both CP and DMD, because of their importance during gait (van der Krogt et al., 2012). For an overview of the complete study design we refer to Table 1.

Table 1Research design.

**Table:** 

**Research design**		
**Main research question**		**Sub question**
**RESEARCH QUESTIONS**		
Does muscle weakness contribute to higher tVAF_1_ during gait in children with CP and boys with DMD compared to TD peers? Or is higher tVAF_1_ in CP related to the underlying brain lesion in CP (expressing the reduction of available DOFs due to the lesion)?		Is weakness of individual muscle groups associated with tVAF_1_ during gait?
**HYPOTHESES**		
Complexity of motor control is influenced by muscle weakness, since muscle weakness could be considered a constraint decreasing the available degrees of freedom during gait		Muscle weakness in the plantar flexors is expected to have the largest influence on the complexity of motor control
**SUBJECTS**		
cerebral palsy *N* = 15	Duchenne muscular dystrophy *N* = 15	Typically-developing children *N* = 15

**Table:** 

**3D gait analysis**	**Maximal voluntary isometric contractions**
**DATA COLLECTION**		
Kinematics and kinetics sEMG of rectus femoris, medial hamstrings, tibialis anterior, gastrocnemius (medial) and gluteus medius	Knee extension Knee flexion Dorsiflexion Plantar flexion
**DATA ANALYSIS**		
Calculation of tVAF_1_ using NNMF on sEMG data from 10 concatenated steps Non-dimensional walking speed	Calculation of torque normalized to bodyweight, averaged over three trials
**Main research question**	**Sub question**
**STATISTICAL ANALYSIS**		
Kruskal-Wallis *H*-test with *post-hoc* Mann-Whitney *U*-test to determine differences in tVAF_1_, walking speed and maximal voluntary contractions between the three groups	Spearman's rank correlation coefficient with classification of Altman

*DOFs, degrees of freedom; N, number; NNMF, non-negative matrix factorization; sEMG, surface electromyography; tVAF_1_, total variance accounted for by one synergy*.

## Materials and methods

In preparation of this study, we performed a power analysis based on a pilot study (Goudriaan et al., 2016) to determine the sample size of the three groups (CP, DMD, and TD; Table 1). The pilot study indicated that for an effect size of *d* = 1.23, α = 0.05, and power (1-β) = 0.80 a minimal sample size of 12 participants per group would be required to test our main hypothesis (GPower 3.1.9, Faul et al., 2007).

### Subjects

We recruited 15 children with CP [median age (interquartile range): 8.9 (2.2)], 15 boys with DMD [8.7 (3.1)], and 15 typical developing (TD) children [8.6 (2.7)] (Table 2). Supplementary Tables 1–3 provide detailed subject characteristics. We asked the children with CP to participate at the time of their routine clinical gait analysis at the Clinical Motion Analysis Laboratory of the University Hospital of Pellenberg (CMAL-Pellenberg) or when they agreed to take part in a large European study, namely the MD-Paedigree project: A Model-Driven Pediatric European Digital Repository, partially funded by the European Commission under P7-ICT-2011-9 program (600932). Inclusion criteria were: (1) diagnosed with bilateral or unilateral CP without signs of dyskinesia, (2) Gross Motor Function Classification System (GMFCS) Levels I-II, (3) no Botulinum Toxin-A treatment within 6 months prior to the assessments, and (4) no history of lower limb surgery.

**Table 2:** Subject characteristics.

	**CP**	**DMD**	**TD**
	**Median (25–75%)**		
**Gender**	**Boys: 7; Girls: 8**	**Boys: 15**	**Boys: 11; Girls: 4**
Diagnosis specifics	H: 8; D:7		
GMFCs level	I: 6; II:9		
Age (years)	8.9 (7.6–9.8)	8.7 (6.8–9.9)	8.6 (7.3–10.0)
Weight (kilograms)	29.0 (22.2–35.7)	23.7 (19.7–33.8)	27.4 (22.6–31.9)
Height (meters)	1.30 (1.20–1.39)	1.16 (1.10–1.29)	1.32 (1.26–1.36)

*CP, cerebral palsy; D, diplegic; DMD, Duchenne muscular dystrophy; H, hemiplegic; TD, typical developing*.

The children with DMD were recruited from the database of the neuromuscular reference center in the University Hospital of Gasthuisberg. If they agreed to participate in MD-Paedigree, we asked them to perform the additional strength measurements needed for the current study. For the DMD children the inclusion criteria were: (1) diagnosed with DMD and (2) no history of lower-limb surgery.

Colleagues and students working at the Clinical Motion Analysis Laboratory of the University Hospital of Pellenberg (CMAL-Pellenberg) assisted with the recruitment of the TD children. The inclusion criteria for the TD children was that they should not have any neurological or neuromuscular problems.

All children were evaluated at the CMAL-Pellenberg. The local ethics committee (Commissie Medische Ethiek KU Leuven) approved this study (S56041), under the Declaration of Helsinki. All the participants' parents or caretakers signed a written informed consent. All participants of 12 years of age or older also signed the informed consent.

### Data collection

We collected gait kinematics, kinetics, and muscle activity data at self-selected walking speed with 3D motion analysis. We used the marker set of the lower limb Plug-in-Gait (PiG) model and marker trajectories were tracked using a 10 to 15-camera VICON system (Nexus 1.8.4. Vicon-UK, Oxford, UK), sampled at 100 Hz. Muscle activity data were collected with surface electromyography (sEMG) bilaterally from the rectus femoris (REF), vastus lateralis (VAL), medial hamstrings (MEH), biceps femoris (BIF), medial gastrocnemius (GAS), soleus (SOL), tibialis anterior (TIA) and the gluteus medius (GLU), with a 16-channel telemetric sEMG system (Zerowire, Cometa, Italy) at 1,000 or 1,500 Hz. Based on the guidelines of Seniam, we attached circular Ag/AgCl electrodes with an area of 1 cm^2^ and an interelectrode distance of 2 cm on the skin (Hermens et al., 1999).

**Figure 1:**
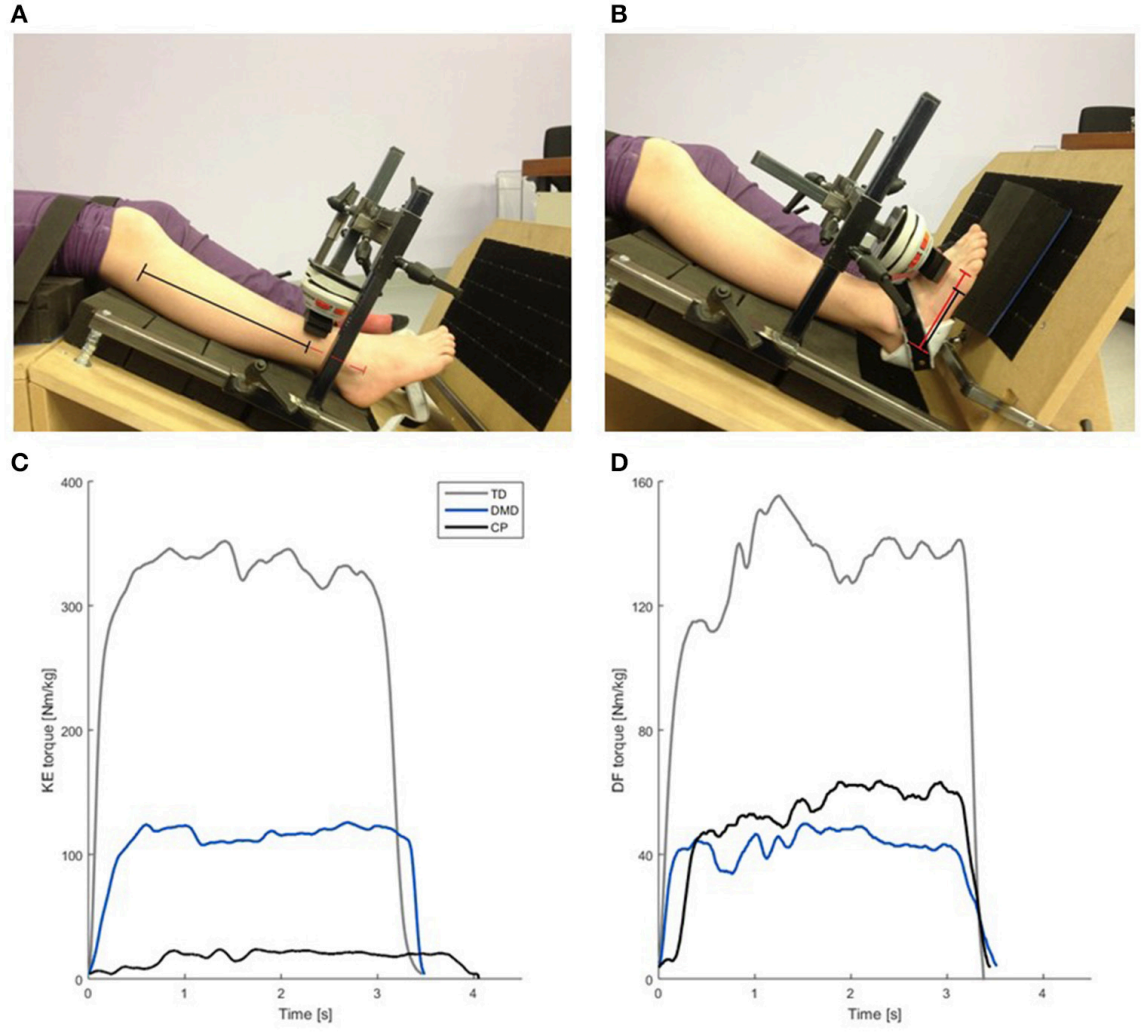
Custom made chair used for the MVIC measurements **(A,B)**, including an example of the normalized net joint torque (Nm/kg) curves collected during the MVIC measurements **(C,D)**. **(A)** Test position for KE MVIC. The black + red lines represent the segment length (fibula head—lower border of lateral malleolus). The black line indicates the moment arm (75% of the segment length). **(B)** Test position for the DF MVIC. The red line represents the segment length (projection of lateral malleolus on lateral border of the foot—distal metacarpal head V). The black line indicates the moment arm (75% of the segment length). **(C)** Normalized knee extension torque (Nm/kg) of a representative KE MVIC of one child with TD (gray), a boy with DMD (blue) and a CP child (black) of similar age. **(D)** Normalized dorsiflexion torque in (Nm/kg) during a representative DF MVIC of the same children as used in **(C)**. Please note the scaling of the axes in **(C,D)** is not the same, due to difference between the knee extensors and the dorsiflexors in torque output. CP, cerebral palsy; DMD, Duchenne muscular dystrophy; DF, dorsiflexion; KE, knee extension; MVIC, maximal voluntary isometric contraction; Nm/kg, Newton meter per kilogram bodyweight; TD, typical developing; s, seconds.

All participants performed MVICs of the knee extensors (KE), knee flexors (KF), dorsiflexors (DF) and plantar flexors (PF) evaluated using a telemetric hand-held dynamometer (HHD) MicroFet® 2 (Hogan Health Industries, West Jordan, UT USA). To decrease compensatory mechanisms and influence of the assessor on MVIC-outcomes, we used a custom-made chair in which the participants were secured with straps around the pelvis and upper legs, and the HHD was fixed to the chair. We placed the HHD at 75% of the segment length (Figure 1) and applied a gravity correction for those MVICs where gravity influenced the output data (KF MVIC and PF MVIC), by subtracting the gravitational torque in rest position from the MVIC-outcomes (Boiteau et al., 1995; Goudriaan et al., 2018). The children first performed one test trial, followed by three actual MVICs, with a duration between 3 and 5 s. Between each trial, the children rested at least 10 s, and between each muscle group, they had a resting period of at least 2 min. During the measurements, the children had visual feedback and were verbally encouraged.

### Data analysis

In the children with CP and the boys with DMD, we first collected the gait analysis data and a standard clinical exam (range of motion, spasticity levels by Modified Ashworth and Tardieu scales, and strength by MMT) and then measured the MVICs by means of dynamometry. Based on the individual child's cooperation during and after the gait analysis and whether the child was fatigued, we decided to collect either bilateral or unilateral MVICs. In case of unilateral MVICs, we always chose the most involved side, based on the outcomes of the standard clinical exam. For the children with CP or DMD, we only included their most involved side in the analyses. In the TD children, MVICs were always collected bilaterally after the gait analysis. Based on the outcomes of the MVICs, we used the weakest leg for further analyses. All available clinical outcome measures are reported in Supplementary Tables 1–3.

We analyzed the sEMG data from five (REF, MEH, TIA, GAS, and GLU) of the eight muscles that were measured during gait. We excluded the VAL, the BIF, and the SOL from all analyses, because their activation patterns (and function) during gait are roughly the same as the REF, MEH, and GAS respectively (Winter, 1987). Also, these five muscles are the most common muscles to be evaluated during standard clinical gait and synergy analyses. For all participants, we selected 10 representative steps. The sEMG signals were filtered with a 6th order Butterworth bandpass filter with cut-off frequencies of 20 and 450 Hz. The signals were rectified and smoothed with a 4th order Butterworth lowpass filter with a frequency of 10 Hz (Shuman et al., 2017). We resampled the filtered sEMG signals of each step at 101 data points, representing 0–100% of a gait cycle. We then concatenated all resampled gait cycles and normalized the signals to the average amplitude of the 10 steps per muscle for each child.

We calculated synergies using non-negative matrix factorization (NNMF) (Lee and Seung, 1999; Ting and Macpherson, 2005; Oliveira et al., 2014; Shuman et al., 2016a) with the NNMF function in MATLAB (The Mathworks Inc., Natick, M.A., 2010) using the following settings: 50 replicates, 1,000 max iterations, 1^*^10^−4^ minimum threshold for convergence, and a 1^*^10^−6^ threshold for completion (Shuman et al., 2016a). NNMF decomposes the sEMG signals into two matrices: *W* containing the synergy weights, which are the weighted contributions of each included muscle to each synergy, and *C*, the synergy activations, such that:

(1) $$\begin{array}{r} sEMG=\left(W_{m*n}*C_{n*t}\right)+error \end{array}$$

In Equation (1), *n* is the number of synergies (one in this study), *m* is the number of muscles (five in this study), *t* is the number of data points (10^*^101 = 1,010 in this study), and *error* is the difference between the measured sEMG data and the reconstructed sEMG signals from the calculated synergies. The *error* value was then used to calculate tVAF as:

(2) $$\begin{array}{r} tVAF_{n}=\;\left(1-\frac{\left[\sum_{j}^{t}\sum_{i}^{m}{\left(error\right)}^{2}\right]}{\left[\sum_{j}^{t}\sum_{i}^{m}{\left(EMG\right)}^{2}\right]}\right) \end{array}$$

From an early age, contractile tissue of the muscles in children with DMD is replaced with fibrofatty tissue (Jansen et al., 2012). Fibrofatty tissue in the muscles might function as an additional lowpass filter (Farina et al., 2002), reducing tVAF_1_ (van der Krogt et al., 2016; Shuman et al., 2017). We therefore calculated the power spectral density (PSD) of the bandpass filtered (20–450 Hz) sEMG signals with the PWELCH function in MATLAB using the following inputs: a window size of 1,024 samples, an overlap of 512 samples, 500 points to use in the Fourier transform, and the sample frequency of the sEMG signals (1,000 or 1,500 Hz). From the PSDs, we calculated the median frequency curves to compare group differences.

Walking speed (in m/s) was extracted from the gait data for each child and converted to a non-dimensional value with the formula of Hof (1996) to determine whether differences in walking speed could explain potential differences in tVAF_1_ between the three groups (Ivanenko, 2005; Shuman et al., 2016a). Force data (in Newtons) from the MVICs was resampled to 100 Hz and the average maximal force out of three trials was calculated (Willemse et al., 2013; Goudriaan et al., 2018). Subsequently, the net joint torque normalized to bodyweight (Nm/kg) was determined for all MVICs for each participant (Supplementary Tables 1–3).

### Statistical analysis

Since the data were not normally distributed, we used non-parametric tests in SPSS (SPSS Inc., Chicago, IL). We used a Kruskal-Wallis *H*-test and a *post-hoc* Mann-Whitney *U*-test with Bonferroni correction (resulting in the critical *p* = 0.005) to determine if there were significant differences in tVAF_1_, non-dimensional walking speed, and MVICs between the three groups (CP, DMD, and TD). The PSD-plots were visually inspected for each muscle. We analyzed the relationship between tVAF_1_ and muscle weakness in each individual muscle group with Spearman's rank correlation coefficients. We used the Altman classification (<0.20 = poor; 0.21–0.40 = fair; 0.41–0.60 = moderate; 0.61–0.80 = good; 0.81–1.00 = very good) to interpret the correlation coefficients (Altman, 1991).

## Results

**Figure 2:**
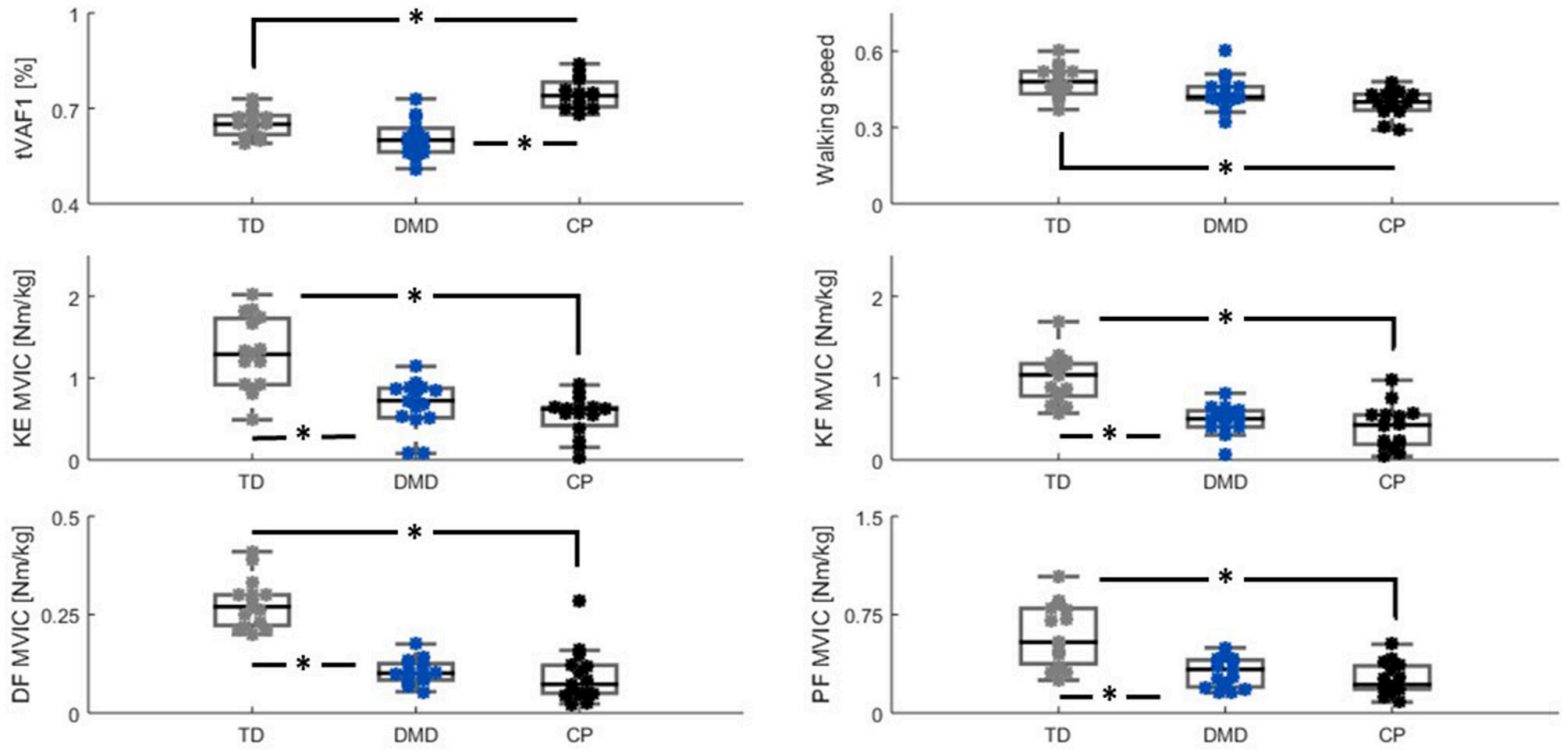
Group differences (TD (gray), DMD (blue) and CP (black)) between tVAF_1_, walking speed, and MVIC-outcomes. Significant differences (*p* ≤ 0.005) based on the Mann-Whitney *U*-test are indicated with ^*^. Please note the scaling on MVIC axes are not the same, due to differences in maximal net joint torque between muscle groups. CP, cerebral palsy; DMD, Duchenne muscular dystrophy; DF, dorsiflexion; KE, knee extension; KF, knee flexion; Nm/kg, Newton meter per kilogram bodyweight; MVIC, maximal voluntary isometric contraction; PF, plantar flexion; tVAF_1_, total variance accounted for by one synergy; TD, typical developing.

In the three study groups (CP, DMD, and TD), all five muscles showed good quality sEMG data for all 10 concatenated steps and could be included in the NNMF analysis, with the exception of the GLU for one of the TD children. The outcomes of the Kruskal-Wallis *H*-Test showed significant group differences for all assessed parameters (all *p* < 0.005). The results of the *post-hoc* Mann Whitney *U*-test on all parameters are plotted in Figure 2.

The tVAF_1_ was significantly higher in the children with CP compared to DMD and TD children (both *p* < 0.005). No significant differences in tVAF_1_ were found between the boys with DMD and the TD children. Median values for tVAF_1_ were 0.74 in CP, 0.60 in DMD, and 0.65 in the TD children. The interquartile ranges (IQRs) were similar for the three groups, 0.09 for the children with CP, and 0.07 for both the boys with DMD and the TD children.

The children with CP and DMD walked slower than the TD children, but this was only significant for the children with CP (*p* < 0.005). Median values and IQRs for non-dimensional walking speed were: 0.40 (0.07) for the CP children, 0.42 (0.05) in the boys with DMD, and 0.48 (0.10) in the TD children.

The TD children were significantly stronger in all four muscle groups than the children in the other two groups (CP and DMD, all *p* < 0.005). The TD children showed more inter-subject variability in MVICs compared to CP and DMD, which was indicated by the larger IQRs. Median values and IQRs in Nm/kg of the knee extensors were: 0.62 (0.26) in CP, 0.72 (0.37) in DMD, and 1.29 (0.83) in TD. For the knee flexors, these values were: 0.43 (0.37) in CP, 0.50 (0.21) in DMD, and 1.04 (0.41) in TD. The dorsiflexors had the lowest MVIC values in all groups, with median values (IQRs) of 0.07 (0.07) in CP, 0.10 (0.04) in DMD, 0.27 (0.08) in TD. For the plantar flexors, median MVIC values of 0.22 (0.19), 0.33 (0.22), and 0.86 (0.45) were found for the CP, DMD, and TD groups, respectively.

**Figure 3:**
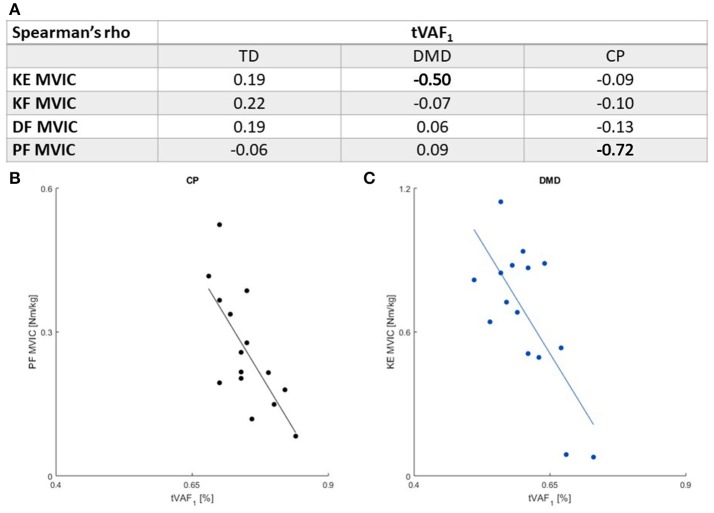
**(A)** Spearman's rank correlation coefficients between MVIC outcomes and tVAF_1_. Moderate or higher correlations (*r* ≤ 0.41) are indicated in bold. There were significant associations between **(B)**. PF MVIC and tVAF_1_ in children with CP and **(C)**. KE MVIC and tVAF_1_ in children with DMD. Please note the scaling of the axes in **(B,C)** are not the same, due to differences in maximal net joint torque. CP, cerebral palsy; DMD, Duchenne muscular dystrophy; DF, dorsiflexion; KE, knee extension; KF, knee flexion; MVIC, maximal voluntary isometric contraction; PF, plantar flexion; TD, typical developing; tVAF_1_, total variance accounted for by one synergy.

Only two moderate-to-high correlations (*r* ≤ 0.41) were found between muscle weakness and tVAF_1_. Increased weakness in the plantar flexors was associated with higher tVAF_1_ in the CP children (*r* = −0.72). In the boys with DMD, weaker knee extensors were associated with higher tVAF_1_ (*r* = −0.50) (Figure 3).

**Figure 4:**
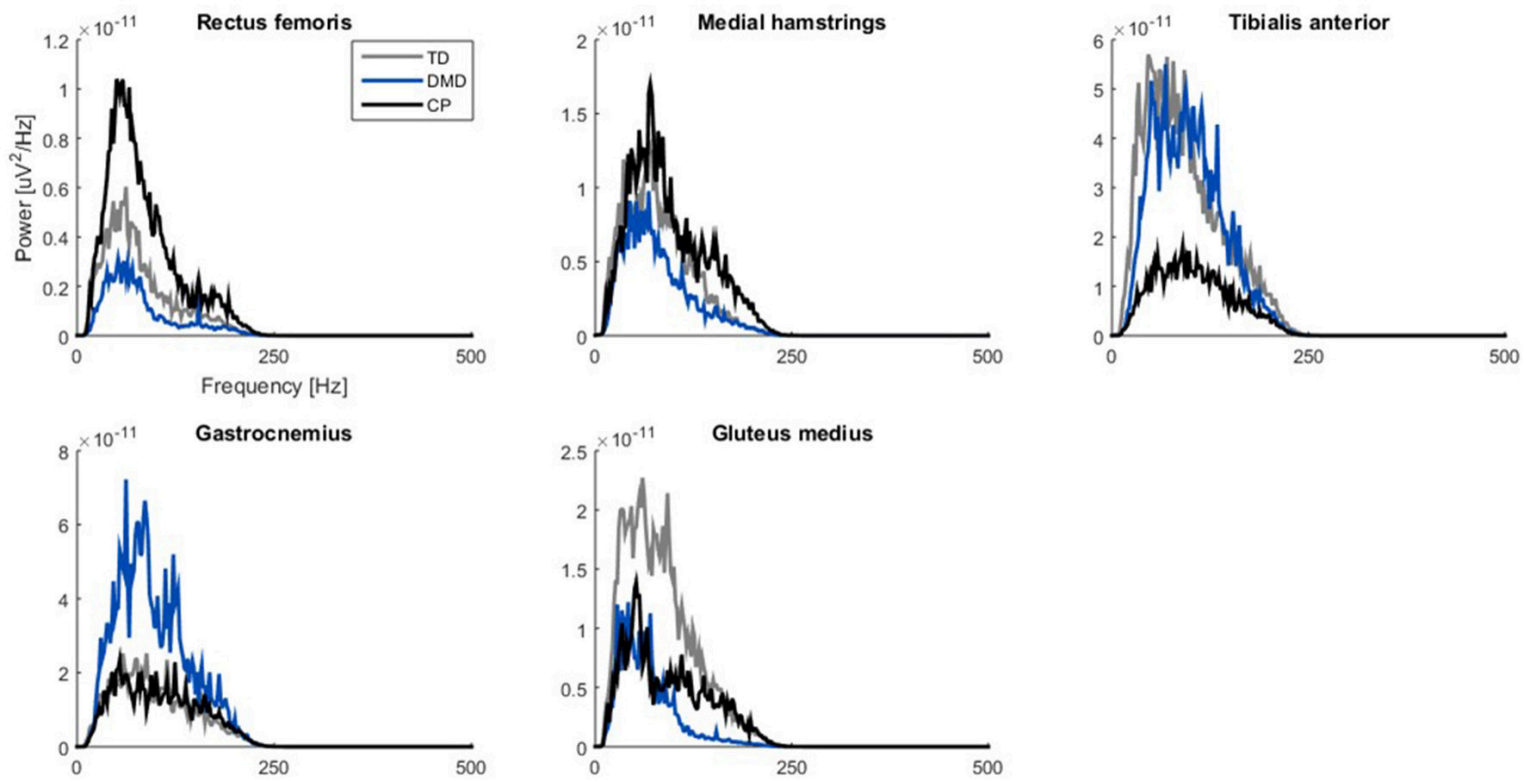
Power spectrum density plots of filtered sEMG signals (20–450 Hz). Median curves are plotted for each group: TD (gray), DMD (blue) and CP (black). Please note the scaling of the axes is not the same, due to differences between muscles. CP, cerebral palsy; DMD, Duchenne muscular dystrophy; Hz, Herz; TD, typical developing; μV, microvolts.

When examining the PSD-plots of the three groups, they showed similar frequency bands, but in DMD the power was lower in the proximal muscle groups (Figure 4).

## Discussion

This study evaluated synergy complexity and strength in two common neuromuscular disorders to explore the impact of the neural and non-neural factors of muscle weakness on neuromuscular control during walking. Due to the differing etiology of these populations, this analysis helps to evaluate which factors serve as constraints to the CNS and influence impaired movement. We hypothesized that muscle weakness contributes to altered synergies and complexity of control during gait in children with CP and DMD, expressed by increased tVAF_1_ during gait compared to TD children. Contrary to this hypothesis, our results suggested that the complexity of control was not influenced by the non-neural constraints of muscle weakness, since tVAF_1_ was not significantly different between the children with DMD and the TD children. However, we confirmed that children with CP had reduced synergy complexity and that muscle weakness in the plantar flexors was related to higher tVAF_1_ during gait. In children with DMD, increased weakness in the knee extensors influenced tVAF_1_, although not enough to result in significantly different tVAF_1_ between DMD and TD.

Similar to previously reported results, tVAF_1_ was higher in the children with CP than in the group of TD children (Steele et al., 2015; Tang et al., 2015; Shuman et al., 2016a). The children with CP walked significantly slower than the TD children, which suggests that the differences in tVAF_1_ between CP and the TD children might be more pronounced than expressed in our results, since a faster walking speed can result in higher tVAF_1_ (Ivanenko, 2005; Shuman et al., 2016a). It could be that tVAF_1_ gives an indirect representation of the child's neural capacity, with a higher tVAF_1_ reflecting a higher level of involvement and increased muscle weakness. This agrees with the findings of Rose and McGill (2005) who determined that muscle weakness in children with CP is largely caused by neural factors. Further, the region on the motor cortex responsible for the distal muscle groups of the lower limb, is closer to the phylogenetic older parts of the brain (Volpe, 2000; Stiles and Jernigan, 2010) and it has been suggested that the older regions of the motor cortex are involved in synergy regulation (Bizzi and Cheung, 2013). Combined with the importance of the plantar flexors during gait, this might explain why we only found a strong correlation between weakness of the plantar flexors and tVAF_1_ during gait and not between tVAF_1_ and the other muscle groups in CP.

We checked if fibrofatty tissue in the muscles of the boys with DMD could act as an additional lowpass filter (Farina et al., 2002; Jansen et al., 2012) thereby reducing tVAF_1_ (van der Krogt et al., 2016; Shuman et al., 2017). In DMD, the proximal muscle groups are more involved than the distal muscle groups, which was also represented in the PSD-plots. While the three groups showed similar frequency bands for all five muscles, the power was lower in the REF, MEH, and GLU muscles in the children with DMD. In children with DMD, fiber type IIb is the first fiber type to degenerate, which will have an influence on the frequency distribution, since these are the fast fibers connected to the motor units with the higher firing frequencies (Stackhouse et al., 2005; Jones et al., 2010).

In the children with DMD, the knee extensors are one of the most involved muscle groups (Sussman, 2002), which could explain the moderate negative correlation between the outcomes of the KE MVIC and tVAF_1_. But, this non-neural weakness of the knee extensors did not sufficiently limit the complexity of control to create a difference in tVAF_1_ between the children with DMD and the TD children. In other words, non-neural weakness appears to be only a small constraint for the CNS with respect to complexity of motor control.

Our results suggest that complexity of motor control, represented by tVAF_1_, might be considered the neural capacity of a child, which could be difficult to alter with current treatments. Although tVAF_1_ measured before treatment has been shown to be associated with changes in gait after treatment (Schwartz et al., 2016), prior research has also demonstrated that there are minimal changes in tVAF_1_ after botulin toxin injections, selective dorsal rhizotomy, and single event multilevel surgeries among children with CP (Oudenhoven et al., 2016; Shuman et al., 2016b).

There are several important limitations in this research. First, we only correlated weakness with tVAF_1_, whereas in children with CP and DMD, other clinical symptoms could have contributed to an increase in tVAF_1_. Steele et al. (2015) determined that a higher level of spasticity and decreased selective motor control were also related to higher tVAF_1_ in children with CP, although to a lesser extent than muscle weakness. Similar to muscle weakness, if a higher tVAF_1_ indicates a higher level of involvement, this would not only be associated with more muscle weakness, but also with spasticity and decreased selective motor control (Ostensjø et al., 2004). In this study we focused on weakness, since DMD provides a comparison group to probe the relative impacts of non-neural factors that contribute to weakness on the results of synergy analysis. However, in children with DMD, other non-neurological symptoms besides muscle weakness are also present, such as decreased passive range motion due to contractures (Sussman, 2002). This only strengthens our conclusion that tVAF_1_ represents the decreased DOFs in the CNS due to the brain lesion and that non-neural constraints have negligible influence on the complexity of motor control.

Further, due to the decreased selective motor control, an increase in level of co-contraction during strength assessments has been reported in children with CP (Mockford and Caulton, 2010). This increase in co-contraction has been suggested to be an important reason for the decrease in maximal torque output during a MVIC (Elder et al., 2003; Stackhouse et al., 2005). However, in a previous pilot study, while using the same protocol to measure MVICs, we determined that the levels of co-contraction were comparable between children with CP and TD children (Goudriaan et al., 2015). Similar results have been reported by Damiano et al. (2000), who determined that, although children with CP had higher levels of co-contraction during knee extension and knee flexion MVICs, this did not influence the outcomes of the MVICs.

Although the plantar flexors are important in maintaining a normal gait pattern, other muscle groups such as the hip abductors also play an important role (van der Krogt et al., 2012). Unfortunately, our MVIC setup did not allow for standardized strength measurements of the hip muscles, thus the influence of weakness in these muscle groups on tVAF_1_ during gait should be analyzed in the future. Finally, tVAF_1_ outcomes in this study were only representative of the five muscles that were included in the analysis. If other muscles were to be analyzed or more muscles included, the value of tVAF_1_ could differ since synergy analyses can be dependent on the number and choice of muscles (Steele et al., 2013). However, it is expected that the relative differences in tVAF_1_ between the three groups (CP, DMD and TD) would be similar.

## Conclusion

The lack of significant differences in tVAF_1_ between boys with DMD and TD children suggests that non-neural muscle weakness has little influence on complexity of motor control during gait. Although, weakness in the plantar flexors was negatively correlated with tVAF_1_ in the children with CP, this is most likely the result of the common underlying cause: alterations in the CNS. Our results imply that despite the predictive value of tVAF_1_ on treatment outcomes, a child's baseline tVAF_1_ (i.e., the child's neural capacity) could be difficult to influence with pre-surgery therapy or may require novel intervention strategies that more directly target neural capacity.

## Author contributions

All authors contributed to the work either to the design, data collection, analysis, interpretation, writing, or editing. KD and MG designed the experiment. Patient recruitment was performed by MVdH, NG, and GM. Data collection was done by MG. BS and KS created the original software for synergy calculation, modifications were made by MG. MG and MVdH performed quality checks on the data. Data analysis was done by MG, BS, and KS. Statistical tests were run by MG. Interpretation of the results was done by KD, KS, BS, and MG. MG and KD wrote the paper, which was edited by KS, BS, MVdH, NG, and GM. The entire process supervised by KD.

### Conflict of interest statement

The authors declare that the research was conducted in the absence of any commercial or financial relationships that could be construed as a potential conflict of interest.
