# Supplementary material for: Non-neural Muscle Weakness Has Limited Influence on Complexity of Motor Control during Gait
Source: Front Hum Neurosci. 2018 Jan 31;12:5. doi: 10.3389/fnhum.2018.00005 (PMC5797794; doi:10.3389/fnhum.2018.00005)
Supplement: Supplementary file 1 [file Table1.PDF]

Supplementary Table 1. Detailed subject characteristics of the children with CP. Dorsi- and plantar flexion passive range of motion were measured with extended knee. Spasticity was scored with the modified Ashworth scale.

	Dx specs	GMFCS	Included side	Gender	Age years	Weight kilogram	Height meters	Leg length meters	Walking speed Non-dim	$tVAF_1$	Knee extension MVIC Nm/kg	Knee flexion MVIC Nm/kg	Dorsi-flexion MVIC Nm/kg	Plantar flexion MVIC Nm/kg	Knee extension PROM degrees	Knee flexion PROM degrees	Dorsi-flexion PROM degrees	Plantar flexion PROM degrees	Knee extension SPAS	Knee flexion SPAS	Plantar flexion SPAS
CP1	D	I	Right	Boy	15.9	49.1	1.71	0.902	0.39	0.79	0.152	0.527	0.082	0.215	-25	normal	-20	normal	2	2	3
CP2	HR	I	Right	Boy	12.2	35.7	1.47	0.760	0.48	0.74	0.573	0.221	0.054	0.216	5	normal	5	normal	1	1	1+
CP3	D	II	Right	Girl	10.2	29.6	1.39	0.731	0.29	0.82	0.627	0.431	0.073	0.179	0	normal	5	normal	1+	1+	2
CP4	D	II	Right	Girl	9.1	57.5	1.49	0.724	0.36	0.84	0.624	0.082	0.050	0.083	0	normal	15	normal	2	1+	2
CP5	D	I	Left	Girl	7.6	20.0	1.20	0.573	0.46	0.70	0.034	0.042	0.025	0.366	0	normal	0	normal	1	1	1+
CP6	HL	II	Left	Boy	8.9	29.0	1.31	0.665	0.36	0.70	0.641	0.552	0.104	0.524	0	normal	20	normal	1+	1+	1+
CP7	D	II	Left	Girl	9.0	24.4	1.31	0.660	0.44	0.75	0.639	0.180	0.045	0.277	5	normal	15	normal	1+	1+	1+
CP8	D	II	Left	Girl	9.3	32.6	1.23	0.680	0.43	0.76	0.621	0.241	0.119	0.118	0	normal	5	normal	3	1	3
CP9	HL	I	Left	Girl	7.0	23.0	1.22	0.588	0.40	0.74	0.916	0.974	0.148	0.257	0	normal	10	normal	0	1	2
CP10	HR	I	Right	Girl	9.8	37.3	1.36	0.712	0.43	0.68	0.839	0.754	0.159	0.417	0	normal	5	normal	0	0	1+
CP11	D	II	Left	Boy	8.5	30.5	1.30	0.662	0.30	0.75	0.376	0.561	0.122	0.386	-5	normal	-5	normal	2	0	1
CP12	HL	II	Left	Boy	7.8	20.4	1.16	0.598	0.42	0.72	0.749	0.542	0.283	0.337	10	normal	20	normal	1+	1	1
CP13	HL	II	Left	Girl	7.7	26.5	1.25	0.605	0.43	0.80	0.564	0.231	0.050	0.149	0	normal	-5	normal	2	N.M.	3
CP14	HR	I	Right	Boy	6.5	22.2	1.20	0.585	0.40	0.74	0.242	0.427	0.056	0.203	0	normal	10	normal	1+	0	2
CP15	HR	II	Right	Boy	6.6	20.4	1.16	0.533	0.40	0.70	0.553	0.149	0.023	0.194	0	normal	10	normal	0	1+	2
25%					7.6	22.2	1.20	0.588	0.36	0.70	0.38	0.18	0.05	0.19							
Median					8.9	29.0	1.30	0.662	0.40	0.74	0.62	0.43	0.07	0.26							
75%					9.8	35.7	1.39	0.724	0.43	0.79	0.75	0.56	0.15	0.37							

Abbreviations in alphabetic order: CP = Cerebral Palsy; Dx specs = diagnosis specifics; GMFCS = Gross Motor Function Classification System; MVIC = maximal voluntary isometric contraction; N.M. = not measured; Nm/kg = Newton meters per kilogram bodyweight; Non-dim = non-dimensional; $tVAF_1$ = total variance accounted for by one synergy; PROM = passive range of motion; SPAS = spasticity;
