# Supplementary material for: Non-neural Muscle Weakness Has Limited Influence on Complexity of Motor Control during Gait
Source: Front Hum Neurosci. 2018 Jan 31;12:5. doi: 10.3389/fnhum.2018.00005 (PMC5797794; doi:10.3389/fnhum.2018.00005)
Supplement: Supplementary file 2 [file Table2.PDF]

Supplementary Table 2. Detailed subject characteristics of the children with DMD. Dorsi- and plantar flexion passive range of motion were measured with extended knee.

	**Included side**	**Gender**	**Age** years	**Weight** kilogram	**Height** meters	**Leg length** meters	**Steroid regimen** Calcort mg/d	**Walking speed** Non-dim	**tVAF$_1$**	**Knee extension MVIC** Nm/kg	**Knee flexion MVIC** Nm/kg	**Dorsi-flexion MVIC** Nm/kg	**Plantar flexion MVIC** Nm/kg	**Knee flexion PROM** degrees	**Knee extension PROM** degrees	**Dorsi-flexion PROM** degrees	**Plantar flexion PROM** degrees
DMD1	Left	Boy	12.4	37.0	1.29	0.650	18	0.41	0.63	0.495	0.639	0.143	0.332	normal	-15	5	normal
DMD2	Left	Boy	9.9	24.2	1.15	0.550	18	0.32	0.72	0.078	0.395	0.103	0.385	normal	-10	10	normal
DMD3	Left	Boy	9.8	28.7	1.31	0.645	21	0.46	0.58	0.878	0.648	0.121	0.353	normal	0	0	normal
DMD4	Left	Boy	9.8	33.8	1.24	0.610	18	0.38	0.67	0.533	0.610	0.127	0.436	normal	0	5	normal
DMD5	Left	Boy	10.4	34.7	1.29	0.647	21	0.42	0.56	1.143	0.571	0.104	0.154	normal	10	0	normal
DMD6	Left	Boy	8.7	23.7	1.16	0.541	18	0.50	0.64	0.885	0.403	0.175	0.389	normal	15	20	normal
DMD7	Left	Boy	8.7	20.4	1.19	0.570	18	0.41	0.59	0.681	0.403	0.101	0.231	normal	0	5	normal
DMD8	Right	Boy	8.8	41.7	1.40	0.738	0	0.36	0.68	0.090	0.066	0.054	0.161	normal	5	0	normal
DMD9	Left	Boy	7.6	20.5	1.12	0.530	15	0.42	0.56	0.847	0.534	0.082	0.497	normal	5	10	normal
DMD10	Right	Boy	6.8	17.0	1.00	0.475	12	0.42	0.61	0.867	0.815	0.068	0.409	normal	10	0	normal
DMD11	Left	Boy	6.8	16.1	1.03	0.475	12	0.51	0.61	0.509	0.502	0.100	0.181	normal	5	5	normal
DMD12	Left	Boy	5.1	19.7	1.08	0.505	15	0.60	0.57	0.724	0.488	0.089	0.417	normal	0	5	normal
DMD13	Left	Boy	6.0	22.4	1.15	0.532	18	0.46	0.60	0.936	0.550	0.077	0.188	normal	5	5	normal
DMD14	Right	Boy	5.7	15.8	1.02	0.485	12	0.43	0.54	0.642	0.302	0.134	0.271	normal	10	20	normal
DMD15	Left	Boy	17.6	32.4	1.35	0.709	18	0.47	0.51	0.817	0.463	0.094	0.267	normal	5	10	normal
25%			6.8	20.1	1.10	0.505	15	0.41	0.56	0.51	0.40	0.08	0.19				
Median			8.7	23.7	1.16	0.550	18	0.42	0.60	0.72	0.50	0.10	0.33				
75%			9.9	33.1	1.29	0.647	15	0.47	0.64	0.88	0.61	0.13	0.41				

Abbreviations in alphabetic order: DMD = Duchenne muscular dystrophy; MVIC = maximal voluntary isometric contraction; Nm/kg = Newton meters per kilogram bodyweight; Non-dim = non-dimensional; tVAF$_1$ = total variance accounted for by one synergy; PROM = passive range of motion;
