# Supplementary material for: Non-neural Muscle Weakness Has Limited Influence on Complexity of Motor Control during Gait
Source: Front Hum Neurosci. 2018 Jan 31;12:5. doi: 10.3389/fnhum.2018.00005 (PMC5797794; doi:10.3389/fnhum.2018.00005)
Supplement: Supplementary file 3 [file Table3.PDF]

Supplementary Table 3. Detailed subject characteristics of the TD children.

	Included side	Gender	Age<br>years	Weight<br>kilogram	Height<br>meters	Leg length<br>meters	Walking speed<br>Non-dim	$tVAF_1$	Knee extension MVIC<br>Nm/kg	Knee flexion MVIC<br>Nm/kg	Dorsi-flexion MVIC<br>Nm/kg	Plantar flexion MVIC<br>Nm/kg
TD1	Left	Boy	15.4	50.4	1.66	0.865	0.43	0.73	1.748	1.271	0.299	0.711
TD2	Left	Boy	11.7	36.2	1.48	0.765	0.44	0.71	2.023	1.692	0.394	0.790
TD3	Left	Boy	10.1	34.4	1.45	0.783	0.42	0.60	1.824	1.039	0.333	0.545
TD4	Left	Girl	10.0	22.6	1.31	0.679	0.46	0.69	1.673	1.208	0.266	0.305
TD5	Left	Boy	8.6	31.9	1.34	0.690	0.54	0.61	1.353	1.089	0.203	0.801
TD6	Left	Boy	8.6	27.5	1.34	0.665	0.37	0.65	1.342	1.176	0.301	0.702
TD7	Left	Boy	7.9	29.9	1.33	0.662	0.55	0.66	1.290	0.868	0.263	1.042
TD8	Left	Girl	9.2	30.3	1.36	0.695	0.41	0.65	0.860	1.160	0.303	0.828
TD9	Right	Boy	7.3	23.0	1.27	0.640	0.52	0.59	1.196	1.120	0.228	0.445
TD10	Left	Girl	8.7	27.3	1.31	0.630	0.52	0.67	1.207	0.638	0.292	0.305
TD11	Right	Boy	8.3	25.6	1.32	0.649	0.48	0.65	1.841	0.882	0.414	0.857
TD12	Right	Boy	6.0	18.1	1.16	0.554	0.52	0.68	0.491	0.571	0.212	0.254
TD13	Left	Girl	7.8	27.4	1.26	0.615	0.51	0.61	0.920	0.662	0.246	0.351
TD14	Left	Boy	5.7	19.0	1.16	0.555	0.46	0.64	0.821	0.814	0.223	0.468
TD15	Right	Boy	6.3	21.9	1.18	0.567	0.60	0.67	0.924	0.767	0.209	0.526
25%			7.3	22.6	1.26	0.615	0.43	0.61	0.92	0.77	0.22	0.35
Median			8.6	27.4	1.32	0.662	0.48	0.65	1.29	1.04	0.27	0.54
75%			10.0	31.9	1.36	0.695	0.52	0.68	1.75	1.18	0.30	0.80

Abbreviations in alphabetic order: MVIC = maximal voluntary isometric contraction; Nm/kg = Newton meters per kilogram bodyweight; Non-dim = non-dimensional; TD = typical developing; $tVAF_1$ = total variance accounted for by one synergy;
